# No Visitors Allowed! The Impact of Covid-19 Restrictions on the Psychosocial Well-being of Nursing Home Residents

**DOI:** 10.1093/geroni/igab046.2718

**Published:** 2021-12-17

**Authors:** Shanae Shaw, Ellen Csikai

**Affiliations:** 1 Alabama State University, Calera, Alabama, United States; 2 The University of Alabama, The University of Alabama, Alabama, United States

## Abstract

The COVID-19 coronavirus pandemic changed life for everyone, but especially for nursing home residents. In March 2020, the Centers for Medicare and Medicaid Services enacted nursing home restrictions in response to the pandemic regarding visitation from outside family/friends and changes to facility activity programs. Despite the public health concern prompting these restrictions to prevent virus spread, these sudden changes affected nursing home residents’ relationships with their spouses/partners. As part of a larger study to identify the nursing home policies and practices that preserve relationships among nursing home residents with spouses/partners, participants shared facilities’ restrictions, social connection practices, and effect of coronavirus restrictions on residents’ relationships with spouses/partners. The study utilized both an online survey (81 respondents) and ten telephone interviews with nursing home social workers in four Southern states. Twenty-eight percent of participants reported that no visitors were allowed; while 25% allowed couples to visit with one another as usual was reported by 25%. The most noted practices to maintain social connections were phone calls, video calls, and ‘window’ visits between residents and families/friends; however, “it’s just not the same. It’s affected them greatly”. The interviews revealed further details about the detrimental effect of the COVID-19 restrictions on nursing home residents’ overall mental health and attachment relationships with spouses/partners. These results highlight the importance of maintaining social connections between residents and spouses/partners. Nursing home social workers can develop policies and practices that enhance relationships and connections under all circumstances and work with other health care team members to ensure implementation.

